# Development of an Immune-Related LncRNA Prognostic Signature for Glioma

**DOI:** 10.3389/fgene.2021.678436

**Published:** 2021-06-14

**Authors:** Yudong Cao, Hecheng Zhu, Jun Tan, Wen Yin, Quanwei Zhou, Zhaoqi Xin, Zhaoping Wu, Zhipeng Jiang, Youwei Guo, Yirui Kuang, Can Li, Ming Zhao, Xingjun Jiang, Jiahui Peng, Caiping Ren

**Affiliations:** ^1^Department of Neurosurgery, Xiangya Hospital, Central South University, Changsha, China; ^2^Changsha Kexin Cancer Hospital, Changsha, China; ^3^Department of Medical Ultrasonics, Seventh Affiliated Hospital of Sun Yat-sen University, Shenzhen, China; ^4^Key Laboratory for Carcinogenesis of Chinese Ministry of Health, School of Basic Medical Science, Cancer Research Institute, Central South University, Changsha, China

**Keywords:** glioma, lncRNA, immune signature, TCGA, risk score

## Abstract

**Introduction:**

Glioma is the most common primary cancer of the central nervous system with dismal prognosis. Long noncoding RNAs (lncRNAs) have been discovered to play key roles in tumorigenesis in various cancers, including glioma. Because of the relevance between immune infiltrating and clinical outcome of glioma, identifying immune-related lncRNAs is urgent for better personalized management.

**Materials and methods:**

Single-sample gene set enrichment analysis (ssGSEA) was applied to estimate immune infiltration, and glioma samples were divided into high immune cell infiltration group and low immune cell infiltration group. After screening differentially expressed lncRNAs in two immune groups, least absolute shrinkage and selection operator (LASSO) Cox regression analysis was performed to construct an immune-related prognostic signature. Additionally, we explored the correlation between immune infiltration and the prognostic signature.

**Results:**

A total of 653 samples were appropriate for further analyses, and 10 lncRNAs were identified as immune-related lncRNAs in glioma. After univariate Cox regression and LASSO Cox regression analysis, six lncRNAs were identified to construct a prognostic signature for glioma, which could be taken as independent prognostic factors in both univariate and multivariate Cox regression analyses. Moreover, risk score was significantly correlated with all the 29 immune-related checkpoint expression (*p* < 0.05) in ssGSEA except neutrophils (*p* = 0.43).

**Conclusion:**

The study constructed an immune-related prognostic signature for glioma, which contributed to improve clinical outcome prediction and guide immunotherapy.

## Introduction

Glioma is one of the most common primary brain tumors and accounts for greater than 70% of malignant brain tumors (Ostrom et al., 2016), presenting only a 5-year survival rate of 30 to 70% in low-grade glioma patients and less than 5% in the most malignant glioblastoma patients (Gousias et al., 2009; Ostrom et al., 2014). Although advances have been made in glioma treatment, including mass surgical resection, radiotherapy, and chemotherapy, the prognosis and survival rate of glioma patients are still unsatisfactory (Stupp et al., 2009; Wu et al., 2019). Unlike traditional therapeutic strategies of curbing cancer cell proliferation and invasion, more and more research reveals the importance of the tumor microenvironment (TME) in glioma development and progression. TME composed of cancer cells and noncancerous cell types is a complex system, including endothelial cells, pericytes, fibroblasts, and immune cells (Quail and Joyce, 2013). As many as 30 to 50% of the cells in gliomas are microglia or macrophages, and tumor-associated microglia and macrophages (TAMs) within the brain tend to be protumorigenic and accumulate as higher as tumor grade (Komohara et al., 2008; Hambardzumyan et al., 2016). Other immune cells, such as dendritic cells, also play an essential role in cancer immune therapy in recent years (Anguille et al., 2014). Therefore, screening reliable immune predictors and prognostic indicators to improve the prognosis of glioma and guide the individual treatment strategies is warranted.

Long noncoding RNAs (lncRNAs) are noncoding RNAs with more than 200 nucleotides in length without significant protein-coding function (Wilusz et al., 2009). Despite their limited expression levels, growing evidence has revealed that lncRNAs could regulate gene expression at epigenetic, transcriptional, and posttranscriptional levels or directly modulate protein activity (Orom et al., 2010; Augoff et al., 2012; Liu et al., 2015; Wang et al., 2016). LncRNAs have been confirmed to play an oncogenic or suppressive role in tumor growth and metastasis including glioma (Sun et al., 2013). For example, lncRNA CASC2 negatively regulates miR-21 to suppress cell growth of glioma, whereas lncRNA CRNDE promotes glioma cell growth and invasion through mTOR signaling (Kang et al., 2019). LncRNA SNHG18 can promote radioresistance of glioma cells by suppressing semaphorin5A16 (Zheng et al., 2016). In addition, lncRNA DANCR has been proved as a diagnostic marker or a potential therapeutic target for the treatment of glioma through regulating miR-135a-5p/BMI1 axis (Feng et al., 2020). Therefore, identifying immune-related lncRNA to predict the prognosis of glioma patients is of great importance for clinical diagnosis and treatment.

In the present study, we applied single-sample gene set enrichment analysis (ssGSEA), Estimation of STromal and Immune cells in MAlignant Tumor tissues using Expression data (ESTIMATE), and Cell type Identification By Estimating Relative Subsets Of RNA Transcripts (CIBERSORT) to classify the glioma patients by immune infiltration degree. Subsequently, we selected immune-related lncRNAs as well as differentially expressed between cancer and normal samples and used the least absolute shrinkage and selection operator (LASSO) Cox regression analysis to construct a prognosis-related risk model. It is hoped that this study will provide promising targets and stimulate new strategies in glioma patients.

## Materials and Methods

### Data Sets and Grouping of Gliomas

The human glioma transcriptome with format of the FPKM (fragments per kilobase of per million) and corresponding clinical data were downloaded from The Cancer Genome Atlas (TCGA) database^1^. Twenty-nine immune data sets including immune cell types, immune-related pathways, and immune-related functions were obtained from the study by Bindea et al. (2013). According to the 29 immune data sets, the ssGSEA was used to calculate enrichment scores for each sample to establish immune-related term enrichment scores in glioma samples using the R packages “GSVA,” “limma,” and “GSEABase.” According to the ssGSEA scores, glioma samples were divided into high and low immune cell infiltration groups using the R package “hclust.”

### Verification of the Immune Grouping

Estimation of STromal and Immune cells in MAlignant Tumor tissues using Expression data and CIBERSORT were applied to validate the group divided by ssGSEA. ESTIMATE is a method that can deduce the stromal and immune cell proportion using gene expression profiles (Yoshihara et al., 2013). Based on this algorithm, tumor purity, ESTIMATE score, immune score, and stromal score of each glioma sample were calculated using “estimate” in R package. Clustering heatmap and statistical map between the two immune groups were shown using “pheatmap” and “ggpubr” in R package. In addition, human leukocyte antigen (HLA) and CD274 [programmed death 1 ligand [PD-L1]) expression were also compared between the two groups to verify the effect of ssGSEA grouping using “ggpubr” and “limma” in R package. CIBERSORT is another approach to characterize 22 types of immune infiltration cell composition using the deconvolution strategy (Newman et al., 2015). The CIBERSORT web tool^2^ was used, and data with *p* < 0.05 were selected for further study. The proportions of immune cell types determined by CIBERSORT between the two groups were compared using the Kruskal–Wallis test to verify ssGSEA grouping again.

### Screen of Immune-Related LncRNAs

Ensembl database^3^ was used to screen lncRNAs. All lncRNAs with false discovery rate (FDR) < 0.05 and | log2FC| ≥ 0.5 were defined as differentially expressed lncRNAs between the high and low immune cell infiltration groups using the “edgeR” package. To identify differentially expressed lncRNAs between the cancer group and the normal groups, gene expression data that included TCGA lower-grade glioma and glioblastoma (GBMLGG) gene expression RNAseq and The Genotype-Tissue Expression (GTEx) gene expression RNAseq were obtained from the UCSC Xena website^4^. The two profiles were recomputed from raw RNA-Seq data by the UCSC Xena project based on a uniform pipeline and shown as log2(*x*+1) transformed RSEM normalized count. After identifying the brain samples in GTEX, quantile normalization of gene expression combining TCGA and GTEx from UCSC Xena was performed using the “normalizeBetweenArrays” function in limma of R (Ritchie et al., 2015). The differentially expressed lncRNAs between the cancer and normal groups were selected using the “limma” package with FDR < 0.05 and | log2FC| ≥ 0.5. The lncRNAs selected in both two analyses were identified as immune-related lncRNA by Venn analysis.

### Construction and Validation of a Prognostic Immune-Related LncRNA Signature

Samples with follow-up time >30 days were kept, and univariate Cox regression analysis of continuous variables was performed by survival package in R with *p* < 0.05 as the criteria to select prognostic immune-related lncRNA. Then, we applied LASSO Cox analysis, a high-dimensional predictor regression method using 10-fold cross-validations, to construct an optimal risk signature model using the “glmnet” R package. The coefficients of the selected lncRNAs were calculated, and a risk score for each glioma patient was calculated using the following formula: risk score = $\sum_{1}^{\text{n}}\text{coefficient}\,\left(\text{lncRNAn}\right)\times \mathrm{expression}\,\left(\text{lncRNAn}\right)$. According to the formula, the glioma patients were sorted into a high-risk group and a low-risk group with the median risk score as the cutoff.

To examine the performance of the prognostic immune-related lncRNA signature, the receiver operating characteristic (ROC) analysis was performed, and the area under the curve (AUC) was calculated using “survivalROC” package in Kaplan–Meier (K-M) analysis used to compare survival between the high- and low-risk groups by the log-rank test. To show the expression patterns of optimal immune-related lncRNAs between the high- and low-risk groups, principal components analysis (PCA) was applied with R using the “scatterplot3d” and “limma” package.

In addition, we also used univariate and multivariate Cox regression analyses to determine whether the signature could predict prognosis independently from clinical parameters, including age, gender, and grade. The grade of glioma was sorted by 2016 World Health Organization (WHO) classification.

### Correlation Between Immune Infiltration and Prognostic Signature

To further explore the relationship between the signature and TME, the correlation between risk scores and immune infiltration calculated by ssGSEA was calculated by Pearson correlation.

### Clinical Correlation and Functional Enrichment Analysis

We explored the relationship between the expression of each lncRNA in the signature and clinical WHO stage by Wilcoxon signed rank test. In addition, immune-related functional annotation (immune response and immune system process) was performed by GSEA to further explore the immune status between the high- and low-risk groups, and *p* < 0.05 was identified as statistically significant.

### Statistical Analysis

All statistical analyses were applied by R version 4.0.2 and corresponding packages. A two-tailed *p* < 0.05 was considered statistically significant, and FDR was calculated using Benjamini–Hochberg methods for multiple corrections to differential expression analyses results (Benjamini and Hochberg, 1995).

## Results

### Construction and Verification of Glioma Groupings

**FIGURE 1:**
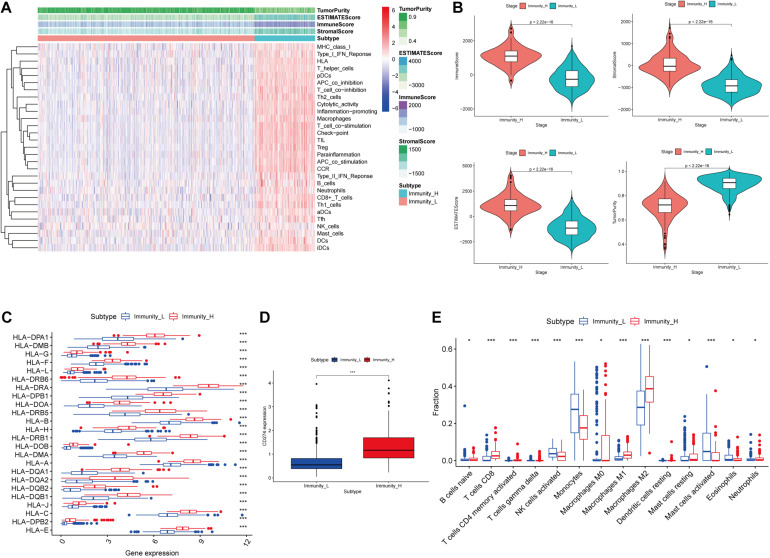
Construction and validation of glioma grouping. **(A)** The display of tumor purity, ESTIMATE score, immune score, and stromal score of each sample gene calculated by ESTIMATE’s algorithm between the high immune infiltration group and the low immune infiltration group. **(B)** The boxplot showed a statistical difference between the two groups in tumor purity, ESTIMATE score, immune score, and stromal score. **(C,D)** The expression of HLA family genes and CD274 between the two groups. **(E)** The proportion difference of immune cells calculated by CIBERSORT method between the two groups. “Immunity_H” and “Immunity_L” represent the high immune cell infiltration group and the low immune cell infiltration group, respectively; *p < 0.05 and ***p < 0.001.

A total of 693 glioma samples were downloaded from TCGA, and all of them were cancer samples. To evaluate infiltration of immune of each sample, ssGSEA was applied, and enrichment scores of the 29 immune-associated gene sets in the TME were obtained. According to the ssGSEA scores, glioma samples were hierarchically clustered into two groups, including the high immune cell infiltration group (*n* = 151) and the low immune cell infiltration group (*n* = 542; Figure 1A).

In the ESTIMATE algorithm, tumor purity, ESTIMATE score, immune score, and stromal score of each glioma sample were determined. Our results showed that tumor purity was significantly lower (*p* < 0.001) in high immune cell infiltration group than that in low immune cell infiltration group; ESTIMATE score, immune score, and stromal score were significantly higher (*p* < 0.001) in high immune cell infiltration group than those in low immune cell infiltration group (Figure 1B). In addition, the expression levels of the HLA family and CD274 (PD-L1) were significantly increasing in the high immune cell infiltration group than those in the low immune cell infiltration group, respectively (*p* < 0.001; Figures 1C,D). In the CIBERSORT algorithm, the high immune cell infiltration group showed higher proportion of immune cells than that in the low immune cell infiltration group (Figure 1E). Based on the above analysis, the immune grouping of the glioma samples was reasonable and feasible for subsequent analysis.

### Identification of LncRNAs Differentially Expressed in Two Classifications

**FIGURE 2:**
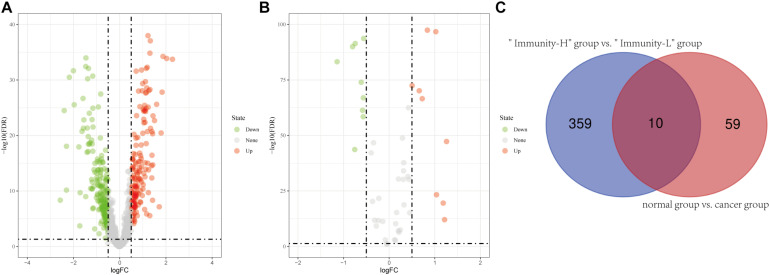
Identification of differentially expressed lncRNAs. **(A)** Differentially expressed lncRNAs between the high immune infiltration group and the low immune infiltration group. **(B)** Differentially expressed lncRNAs between the cancer group and the normal group. **(C)** The intersection of differentially expressed lncRNAs is shown in the Venn diagram. “Immunity_H” and “Immunity_L” represent the high immune cell infiltration group and the low immune cell infiltration group, respectively.

In the 693 glioma samples from TCGA downloaded from the official website, 369 lncRNAs were differentially expressed between the high and low immune cell infiltration groups, including 179 up-regulated lncRNAs and 190 down-regulated lncRNAs in the high immune cell infiltration group (Figure 2A). We obtained 702 glioma samples (697 cancer samples and 5 paracancerous samples) and 1,152 normal brain samples on the UCSC Xena website. After merging the two databases from the UCSC Xena website, we identified 69 differentially expressed lncRNAs between the cancer and normal groups, including 34 up-regulated lncRNAs and 35 down-regulated lncRNAs in the cancer group compared to the normal group (Figure 2B). A two-way Venn analysis was then applied to select lncRNAs which were differentially expressed in both the high immune cell infiltration group compared with the low immune cell infiltration group and the cancer group compared with the normal group. Ultimately 10 lncRNAs were identified as immune-related lncRNAs in glioma (Figure 2C).

### Construction and Validation of Immune-Related LncRNA Prognostic Signature

**FIGURE 3:**
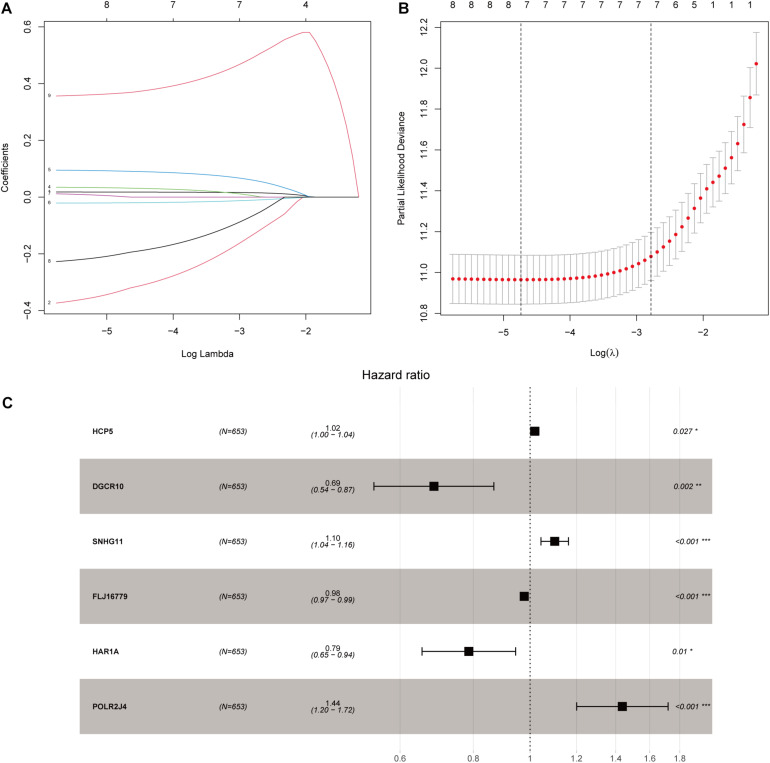
Construction of an immune-related lncRNA prognostic risk score model. **(A)** LASSO Cox regression analysis of the nine prognostic lncRNAs. **(B)** Ten-round cross-validation was conducted for the optimal penalty parameter lambda. **(C)** Multivariate Cox regression analysis of immune-related lncRNAs. *p < 0.05, **p < 0.01, and ***p < 0.001.

**FIGURE 4:**
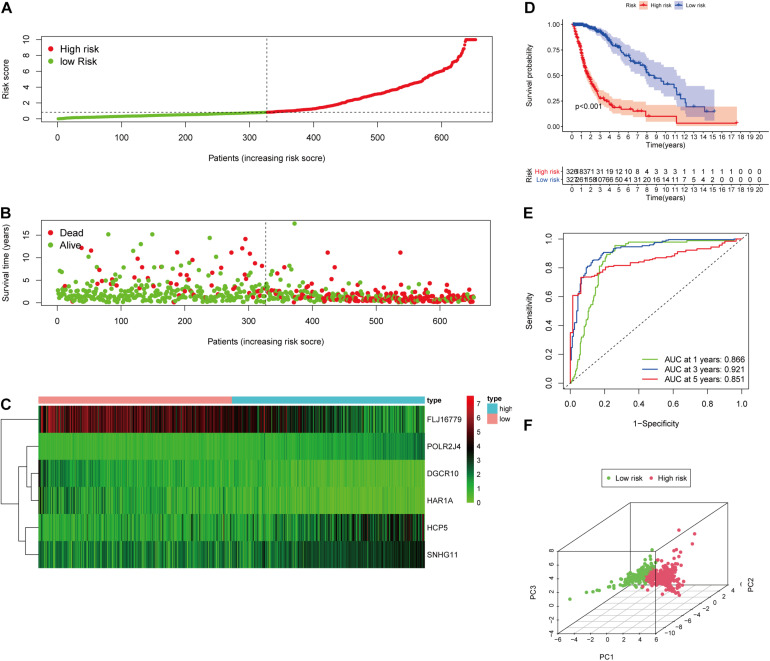
Validation of the immune-related lncRNA prognostic risk score model. **(A)** The distribution of risk score. **(B)** The distribution of patients’ survival time and status. **(C)** Heatmap of selected six immune-related lncRNAs of the classifier. **(D)** Kaplan–Meier curves of high-risk and low-risk score groups. **(E)** Time-dependent ROC analyses of the identified immune-related risk signature. **(F)** PCA analysis based on six survival-related immune lncRNAs.

After screening data with eligible survival information, a total of 653 samples were available for further analyses. Univariate Cox regression analysis was used to screen lncRNA associated with the glioma patients’ overall survival, and nine lncRNAs were selected. Then these nine lncRNAs were entered for LASSO regression analysis and multivariate Cox regression analysis (Figures 3A,B). Ultimately, six lncRNAs (HCP5, DGCR10, SNHG11, FLJ16779, HAR1A, and POLR2J4) were selected in this prognostic signature (Table 1) and the contribution of lncRNAs in the calculation formula is shown in Figure 3C. Therefore, a prognostic prediction model was developed based on the six lncRNAs as follows: risk score = (0.0183) × EXP_HCP5_−(0.3778) × EXP_DGCR10_ + (0.0968) × EXP_SNHG11_−(0.0229) × EXP_FLJ16779_−(0.2407) × EXP_HAR1A_ + (0.3625) × EXP_POLR2J4_. According to the risk score, glioma samples were divided into a low-risk group and a high-risk group with a median risk score as the threshold. HCP5, SNHG11, and POLR2J4 were highly expressed in the high-risk group, whereas DGCR10, FLJ16779, and HAR1A were highly expressed in the low-risk group (Figure 4C). The distribution of risk score and survival status is illustrated in Figures 4A,B.

**TABLE 1:** The expression levels of these six lncRNAs.

ID	Coefficient	HR	HR.95L	HR.95H	*p* value
HCP5	0.0183	1.0185	1.0021	1.0352	0.0273
DGCR10	−0.3778	0.6854	0.5415	0.8674	0.0017
SNHG11	0.0968	1.1017	1.0436	1.1629	0.0005
FLJ16779	−0.0229	0.9773	0.9667	0.9881	4.10e-05
HAR1A	−0.2407	0.7860	0.6541	0.9447	0.0103
POLR2J4	0.3625	1.4369	1.2007	1.7196	7.61e-05

To evaluate the prediction model, the K-M curve revealed that the patients in the high-risk group showed a shorter survival time or lower survival probability compared to the low-risk group (Figure 4D) with 1-, 3-, and 5-year AUC values of 0.866, 0.921, and 0.851, respectively (Figure 4E). The PCA result of the six immune-related lncRNAs is shown in the figure and indicated a significant distinction of the samples after risk score clustering between precorrection and postcorrection (Figure 4F).

### Independent Prognostic Analysis of the Immune-Related LncRNA Prognostic Signature

**FIGURE 5:**
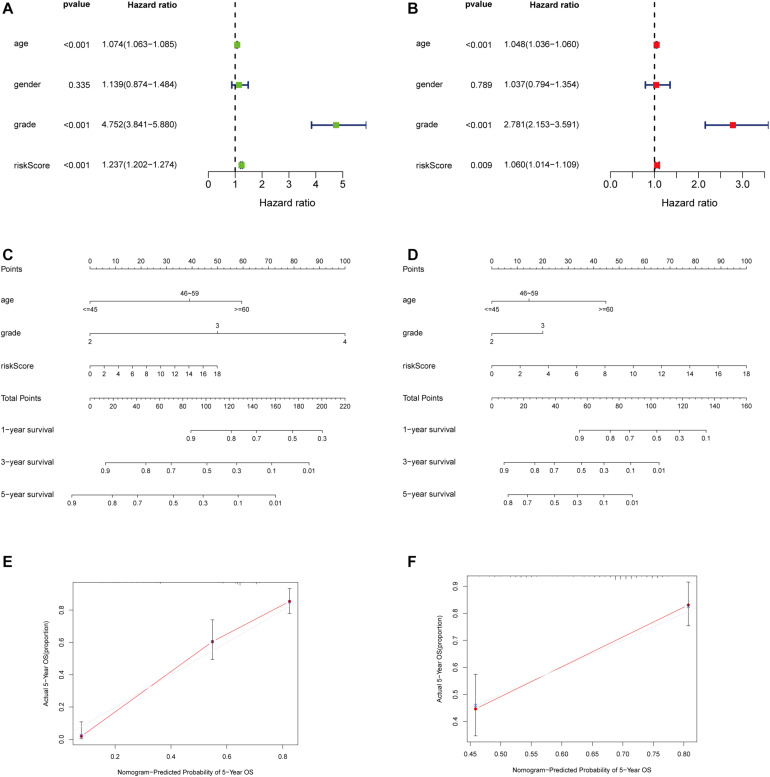
Independent prognostic analysis of the immune-related lncRNA prognostic signature. The univariate **(A)** and multivariate **(B)** Cox regression analysis of risk score, age, gender, and grade. A nomogram to quantitatively predict 1-, 3-, and 5-year survival for all the glioma patients **(C)** and non–glioblastoma multiforme patients **(D)** Calibration curves of the nomogram model for showing the consistency between predicted and actual survival in all the glioma patients **(E)** and non–glioblastoma multiforme patients **(F)**.

Considering that the prognosis of patients with glioma is associated with clinical characteristics such as age, gender, and pathological stage, univariate and multivariate Cox regression analyses were conducted. The results showed that the immune-related lncRNA prognostic signature could be taken as independent prognostic factors, as well as age and grade in both univariate and multivariate Cox regression analyses (Figures 5A,B). The contribution of each independent factor is presented in the nomogram (Figures 5C,E), and it revealed that the grade was the leading factor for predicting nomogram. Then we divided the glioma samples into glioblastoma multiforme (grade 4) and non–glioblastoma multiforme (grades 2 and 3). The nomogram in non–glioblastoma multiforme showed that risk score was the leading predicted factor with the grade as inferior impact (Figures 5D,F).

### Correlation Between Immune Checkpoint Expression and the Risk Score

**FIGURE 6:**
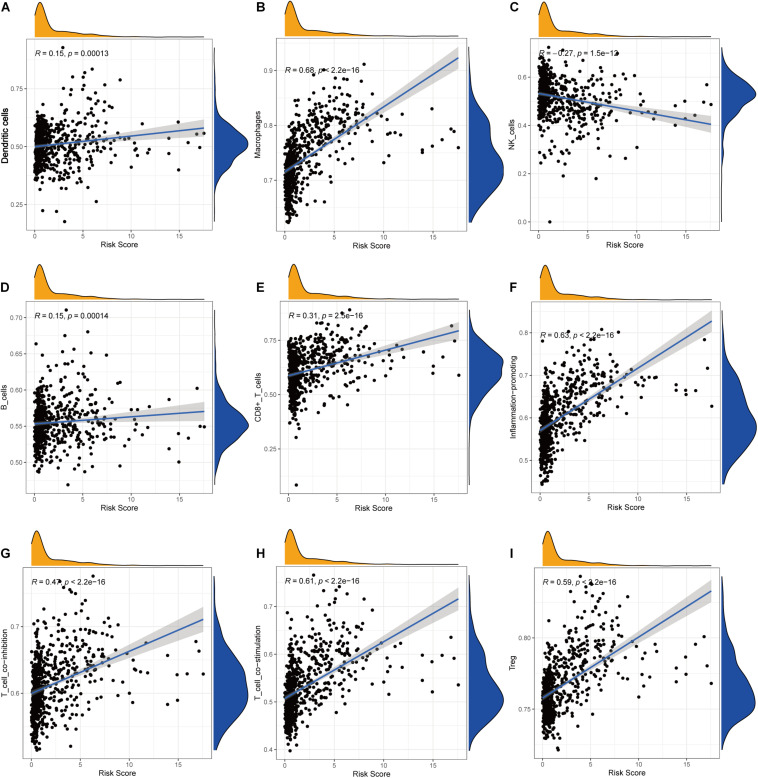
Correlations between the risk score and infiltration abundances of immune cells and immune-related functions. **(A)** Dendritic cells, **(B)** macrophages, **(C)** NK cells, **(D)** B cells, **(E)** CD8^+^ T cells, **(F)** inflammation promoting, **(G)** T-cell coinhibition, **(H)** T-cell costimulation, **(I)** Treg.

Pearson correlation analysis between immune checkpoint expression and the risk score revealed that risk score was significantly correlated with all the 29 immune-related checkpoint expression (*p* < 0.05) in ssGSEA except neutrophils (*p* = 0.43). The relationship is partly displayed in Figure 6.

### Clinical Correlation and GSEA Functional Enrichment Analysis

**FIGURE 7:**
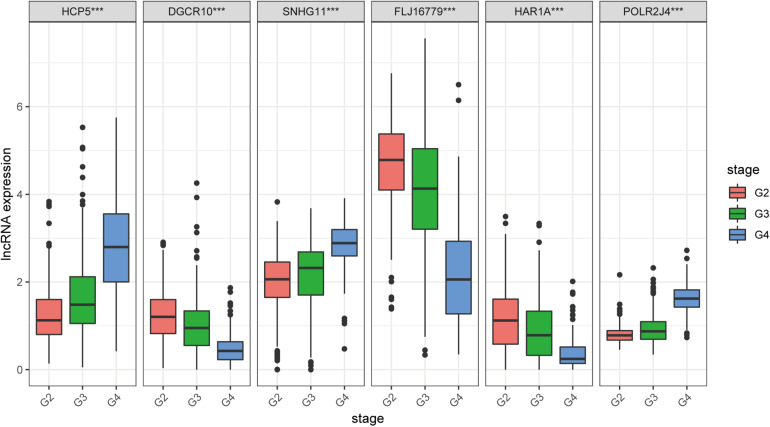
Expression profile of six immune-related lncRNAs in the signature with different glioma grades.

**FIGURE 8:**
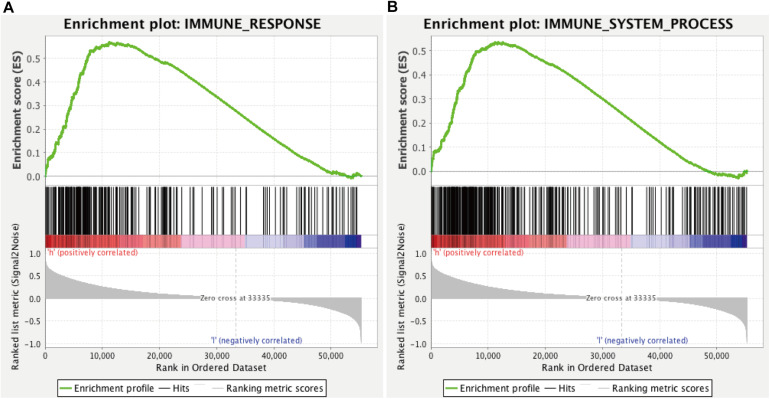
GSEA for comparing immune response **(A)** and immune system process **(B)** between low- and high-risk groups.

We found that all lncRNAs in the signature were significantly different in different grades (Figure 7). In addition, GSEA suggested that immune response term and immune system process term significantly enriched in the high-risk group compared to the low-risk group (Figures 8A,B).

## Discussion

Glioma is the most common primary cancer of the central nervous system. Despite advances in conventional therapy, the prognosis for most glioma patients remains dismal. Nowadays, increasing insight into immunotherapy suggests it may be recognized as an effective treatment alternative. Immunotherapy that aims at stimulating a specific and sustained antitumor response is taken as a promising therapeutic approach. Immunomonitoring can track the effects of immunotherapy upon the patient’s immune system and accelerate the development of immunotherapeutic agents. Therefore, investigating potential biomarkers of clinical benefit that can efficiently reflect treatment efficacy is one of the primary goals of immunomonitoring in glioma immunotherapy trials (Lamano et al., 2016). Thus, in the present study, we constructed a 6-lncRNA prognostic signature related to immune infiltration.

For the first time, immune-related lncRNAs in glioma were identified by screening differentially expressed lncRNAs between the high and low immune cell infiltration groups, which was divided by ssGSEA and verified by ESTIMATE, the expression of HLA and CD724, and the algorithm of CIBERSORT. Similarly, the study by Shen et al. (2020) on immune-related lncRNA prognostic signature for breast cancer also identified immune cell infiltration group by ssGSEA and verified the groups by ESTIMATE and CIBERSORT, which confirmed the feasibility of the methods further, whereas previous studies in glioma identified immune-related lncRNAs by GSEA database or the molecular signature database. In addition, compared to previous studies, the lncRNAs in our signature were not only immune-related but also differentially expressed between the cancer and normal groups (Tian et al., 2020; Xia et al., 2021). In this study, our signature achieved a satisfactory level of 1-, 3-, and 5-year AUC (0.866, 0.921, and 0.851, respectively), which outperformed Xia and colleagues’ and Tian and colleagues’ studies’ 3-year AUC and was comparable with Tian and colleagues’ study’s 1- and 5-year AUCs. Moreover, we established a predicting nomogram combining age and grade to predict the survival of glioma patients more accurately and intuitively. When the samples were divided into glioblastoma (grade 4) and non–glioblastoma (grades 2 and 3), risk score was the dominant factor in the nomogram, indicating risk score was an excellent prognostic factor.

Long noncoding RNAs can be used as biomarkers to classify and predict tumors because they can display characteristic tissue-specific and cell-type–specific expression patterns (Deveson et al., 2017). Increasing evidence has shown that a specific lncRNA plays a role in the onset and progression of various cancers, as well as tumorigenesis and progression in glioma (Huarte, 2015; Bhan et al., 2017). In our study, we extracted nine immune-related lncRNAs correlated with prognosis and differentially expressed in the cancer group compared to the normal group at the same time. After LASSO analysis and multivariate Cox regression analysis, six immune-related lncRNAs were selected to construct a prognostic signature. Most of them have been reported to be related to immune or participate in immune regulation in previous studies. HCP5 was mainly expressed in immune system cells and had an effect on autoimmunity (Li et al., 2018). Researches have indicated that HCP5 acted as an oncogene in glioma, and the expression of HCP5 increased with the level of grade of glioma (Zou and Chen, 2021). It is also reported that knockdown of HCP5 can inhibit proliferation, cell migration, and invasion, so as to promote apoptosis of glioma cells (Teng et al., 2016). SNHG11 was confirmed to express highly in glioblastoma compared to normal brain, and it could promote proliferation, migration, and invasion via epithelial–mesenchymal transition by sponging miR154-5p (Geng et al., 2020). These findings are consistent with our findings that HCP5 and SNHG11 were highly expressed in the high-risk group. POLR2J4 has been reported as a composition of signature to predict the survival of cirrhotic hepatocellular carcinoma and recurrence-free hepatocellular carcinoma (Gu et al., 2019; Ma and Deng, 2019). It was also found to be a predictor of the risk for site-specific metastasis of breast cancer (Park et al., 2020). FLJ16779 was implicated in gastric carcinogenesis and progression via modulating energy metabolism (Wang et al., 2020). HAR1A was expressed specifically in Cajal–Retzius neurons in the developing human neocortex, and a previous study reported that HAR1A could act as a prognostic marker for isocitrate dehydrogenase mutant glioma (Pollard et al., 2006; Shi et al., 2019; Chen et al., 2020). However, no published studies have reported biological functions of DGCR10 so far, and further studies are needed to investigate its molecular characteristics.

Cancer tissues consist of not only malignant neoplastic cells but also immune cells, fibroblasts, endothelial cells, and an abundant collection of cytokines, chemokines, growth factors (Bremnes et al., 2011). Those components and their complicated interaction form the TME, and the various cellular compartments of the TME can critically regulate tumorigenesis, which is essential not only to tumor initiation, malignant progression, and metastasis but also to response to therapy (Klemm and Joyce, 2015). In TME, immune cells are the predominant host cells that are recruited to and activated (Bremnes et al., 2011). In myeloid lineage, TAM inhibition effects on blocking gliomagenesis and activated TAMs have confirmed the ability to regulate glioma stem cell pools within the brain. What is more, because of the plasticity of TAM, it may be feasible to develop strategies to reeducate macrophages to specifically adopt antitumor phenotypes in brain tumors, which are likely to be new immunotherapy methods. In lymphoid lineage, accumulated studies demonstrated that reprogramming of immunosuppressive T-cell subsets might boost antitumor immune responses in glioma or other brain tumors (Quail and Joyce, 2017). It is also an emerging field in cancer therapy to enhance T-cell activation via enabling costimulation primary in gliomas or brain metastases (Fecci et al., 2014; Cohen and Kluger, 2016). In this study, we found that six-lncRNA prognostic signature for glioma was associated with the infiltration of immune cell subtypes, which further verified the signature.

There were some limitations to the present study. First, it was a retrospective study, and prospective cohort studies are needed to further validate the results. Second, the biological functions of the six identified lncRNAs need comprehensive exploration and should be fully elucidated in *in vitro* and *in vivo* experiments, especially in terms of immune infiltration.

In conclusion, our study established a reliable immune-related prognostic signature. With further prospective validation, the signature may become therapeutic targets and offer biological information for personal treatment of glioma.

## Data Availability Statement

Publicly available datasets were analyzed in this study. This data can be found here: the TCGA website (https://portal.gdc.cancer.gov) and the UCSC Xena website (https://xena.ucsc.edu/).

## Author Contributions

XJ and JP conceived and designed the study. YC and JP wrote the manuscript. CR, JT, WY, QZ, ZX, and ZW analyzed the results. HZ, ZJ, YG, YK, CL, and MZ performed the image visualization. All authors contributed to the article and approved the submitted version.

## Conflict of Interest

The authors declare that the research was conducted in the absence of any commercial or financial relationships that could be construed as a potential conflict of interest.

## Footnotes

1 https://portal.gdc.cancer.gov

2 https://cibersort.stanford.edu/

3 https://www.ensembl.org/

4 https://xena.ucsc.edu/
